# Digenic *DUOX1* and *DUOX2* Mutations in Cases With Congenital Hypothyroidism

**DOI:** 10.1210/jc.2017-00529

**Published:** 2017-06-16

**Authors:** Zehra Aycan, Hakan Cangul, Marina Muzza, Veysel N. Bas, Laura Fugazzola, V. Krishna Chatterjee, Luca Persani, Nadia Schoenmakers

**Affiliations:** 1 Division of Paediatric Endocrinology, Dr. Sami Ulus Woman Health and Children Research Hospital, 06080 Ankara, Turkey; 2 Department of Medical Genetics, Istanbul Medipol University, International School of Medicine, 34810 Istanbul, Turkey; 3 Endocrine Unit, Fondazione Istituto di Ricovero e Cura a Carattere Scientifico (IRCCS) Ca’ Granda Policlinico, 20122 Milan, Italy; 4 Department of Pathophysiology and Transplantation, University of Milan, 20122 Milan, Italy; 5 Division of Endocrinology and Metabolism, Istituto di Ricovero e Cura a Carattere Scientifico (IRCCS) Istituto Auxologico Italiano, 20149 Milan, Italy; 6 University of Cambridge Metabolic Research Laboratories, Wellcome Trust–Medical Research Council Institute of Metabolic Science, Addenbrooke's Hospital, Cambridge CB2 0QQ, United Kingdom; 7 Department of Clinical Sciences and Community Health, University of Milan, 20122 Milan, Italy

## Abstract

**Context:**

The DUOX2 enzyme generates hydrogen peroxide (H_2_O_2_), a crucial electron acceptor for the thyroid peroxidase–catalyzed iodination and coupling reactions mediating thyroid hormone biosynthesis. *DUOX2* mutations result in dyshormonogenetic congenital hypothyroidism (CH) that may be phenotypically heterogeneous, leading to the hypothesis that CH severity may be influenced by environmental factors (*e.g.*, dietary iodine) and oligogenic modifiers (*e.g.*, variants in the homologous reduced form of NAD phosphate-oxidase DUOX1). However, loss-of-function mutations in *DUOX1* have not hitherto been described, and its role in thyroid biology remains undefined.

**Case Description:**

We previously described a Proband and her brother (P1, P2) with unusually severe CH associated with a *DUOX2* homozygous nonsense mutation (p.R434*); P1, P2: thyrotropin >100 µU/mL [reference range (RR) 0.5 to 6.3]; and P1: free T4 (FT4) <0.09 ng/dL (RR 0.9 to 2.3). Subsequent studies have revealed a homozygous *DUOX1* mutation (c.1823-1G>C) resulting in aberrant splicing and a protein truncation (p.Val607Aspfs*43), which segregates with CH in this kindred.

**Conclusion:**

This is a report of digenic mutations in *DUOX1* and *DUOX2* in association with CH, and we hypothesize that the inability of DUOX1 to compensate for DUOX2 deficiency in this kindred may underlie the severe CH phenotype. Our studies provide evidence for a digenic basis for CH and support the notion that oligogenicity as well as environmental modulators may underlie phenotypic variability in genetically ascertained CH.

## Teaser

Whole-exome sequencing in siblings with a known homozygous DUOX2 mutation and unusually marked congenital hypothyroidism (CH) identified the human homozygous DUOX1 mutation, segregating with CH.

Congenital hypothyroidism (CH), due to dyshormonogenesis, occurs due to defective thyroid hormone biosynthesis in a structurally normal gland, and causes include mutations in the reduced form of NAD phosphate (NADPH)-oxidase *DUOX2*, which generates the hydrogen peroxide (H_2_O_2_) required for the organification of iodide. *DUOX2* is contiguous with *DUOX1*, which encodes an additional thyroidal NADPH-oxidase on the long arm of chromosome 15, and their respective *DUOXA* maturation factor genes occupy the *DUOX* intergenic region [Supplemental Fig. 1(A)]. The DUOX1 and DUOX2 proteins exhibit 83% sequence homology; however, DUOX2 is thought to be the dominant isoenzyme in the thyroid, as evidenced by its higher thyroidal expression levels and the observations that human mutations in both *DUOX2* and *DUOXA2*, but not *DUOX1*, have been implicated in CH. Additionally, in murine models, only *DUOX2* loss of function is associated with hypothyroidism; thus, the role of DUOX1 in thyroid biology remains unclear (1).

*DUOX2* mutations usually cause transient CH or permanent CH with partial iodide organification defect. Permanent and transient CH may result from both mono- and biallelic mutations, and phenotypic heterogeneity may occur with similar mutations (2). The mechanisms modulating disease severity are unclear and may include genetic or epigenetic factors and environmental contributors, *e.g.*, iodine intake. Because DUOX1 also generates H_2_O_2_ in the thyroid, it has been suggested that this isoenzyme may undergo variable upregulation to compensate for the DUOX2 deficiency, although no naturally occurring *DUOX1* functional variants have hitherto been described.

We previously reported two Probands harboring a homozygous, known pathogenic nonsense mutation in *DUOX2* (p.R434*), both of whom exhibited uncharacteristically severe CH (3). Whole-exome sequencing in this kindred detected digenicity for a homozygous essential splice site *DUOX1* mutation (c.1823-1G>C) in affected individuals, found to be pathogenic *in vitro* and likely contributing to the phenotypic severity.

## Materials and Methods

All investigations were ethically approved and/or clinically indicated, being undertaken with patient or parental consent.

### Biochemical measurements

Hormone measurements were made using local automated assays.

### Molecular genetic studies

Detailed methods for performing and analyzing data from whole-exome sequencing and Sanger sequencing of the *DUOX1* variant are provided in Supplemental Material.

### *In vitro* characterization of the DUOX1 splice site mutation

RNA extracted from peripheral leukocytes was reverse transcribed, and complementary DNA was polymerase chain reaction amplified using primers spanning translated exons 14–18, purified, and directly sequenced (Supplemental Material).

## Results

### Clinical and biochemical features

**Figure 1.**
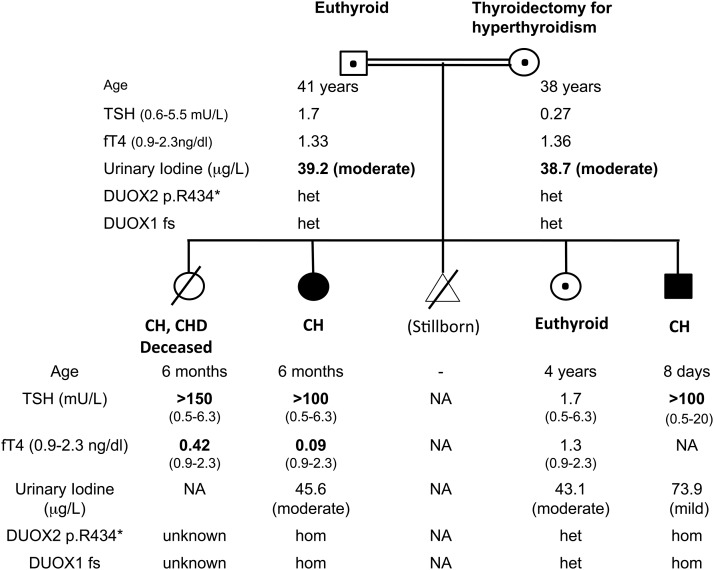
Pedigree diagram summarizing clinical phenotype and genotype. Black: homozygotes for the *DUOX2* and *DUOX1* mutations; central black dot: heterozygotes for the two mutations. The degree of iodine deficiency on the spot urinary measurement is classified according to World Health Organization criteria. DUOX1 fs, DUOX1 p.Val607Aspfs*43; het, heterozygous; hom, homozygous.

The patients’ clinical details have previously been published (3) (Fig. 1). Briefly, three patients with CH were born to consanguineous Turkish parents; the first (female) was diagnosed aged 6 months with thyrotropin (TSH) >150 µU/mL [reference range (RR) 0.5 to 6.3] and FT4 0.42 ng/dL (RR 0.9 to 2.3), but subsequently died due to congenital heart disease. The second (female, P1) presented aged 6 months with growth retardation (height 56 cm, <3rd centile, weight 5.6 kg, <3rd centile), somnolence, and constipation. She had coarse facial features, macroglossia, and severe biochemical hypothyroidism [TSH >100 µU/mL (RR 0.5 to 6.3) and FT4 <0.09 ng/dL (RR 0.9 to 2.3)] and has severe learning difficulties aged 12 years. Thyroid ultrasound aged 3 years demonstrated a normally located thyroid gland (right lobe: 11 × 9 mm; left lobe: 13 × 8 mm), and more quantitative ultrasonography aged 11 years confirmed a normal thyroid volume (right lobe: 34 × 14 × 12 mm; left lobe: 32 × 13 × 14 mm; isthmus: 3.5 mm), although this may have been influenced by the fact that she was on levothyroxine treatment. Her younger brother (P2), who exhibited umbilical hernia, was diagnosed aged 8 days, with goitre and TSH >100 µU/mL (RR 0.5 to 20). Treatment was commenced with 25 µg levothyroxine per day (10 µg/kg/d); however, aged 1 month, TSH remained elevated despite good treatment compliance, suggesting severe CH [TSH 91 µU/mL (RR 0.5 to 9) and FT4 1.2 ng/dL (RR 0.9 to 2.3)], and levothyroxine dose was rapidly increased to 50 µg/d. Thyroid ultrasonography demonstrated a normally located, diffusely hyperplastic gland. Both children ultimately required significant doses of l-thyroxine (87.5 µg, 1.9 micrograms/kg/d, P1 aged 12 years; 75 µg, 3.1 µg/kg/d, P2 aged 5.75 years). Their sister (S1) was unaffected aged 4 years [TSH 1.7 µU/mL (RR 0.5 to 6.3); FT4 1.3 ng/dL (RR 0.9 to 2.3)].

Their father was euthyroid [TSH 1.7 µU/mL (RR 0.6 to 5.5); FT4 1.33 ng/dL (RR 0.9 to 2.3)], and their mother, who had previously undergone thyroidectomy for autoimmune hyperthyroidism, was euthyroid on levothyroxine treatment [TSH 0.27 µU/mL (RR 0.6 to 5.5); FT4 1.36 ng/dL (RR 0.9 to 2.3); anti–thyroid peroxidase antibodies 51 IU/mL (RR 0 to 35)]. Her obstetric history also included two abortions and a hydatidiform mole. She had taken Propisyl for hyperthyroidism during her pregnancies with P2, and S1 but not P1.

### Molecular genetic studies

A previously described homozygous *DUOX2* nonsense mutation (c.1300C>T, p. R434*) had initially been identified in P1 and P2, for which their parents and unaffected sibling were heterozygous (Fig. 1) (3). DNA was not available from the deceased sibling.

The severity of the CH prompted investigation for an additional genetic mutation using whole-exome sequencing in P1 and P2. In addition to coding regions, significant intronic sequences were covered using this technique, enabling detection of a homozygous essential splice site change in *DUOX1* (c.1823-1G>C), at the intron 14/exon 15 boundary, validated by Sanger sequencing in both cases. This was absent from 400 ethnically matched control chromosomes and normal genome datasets [dbSNP, Exome Aggregation Consortium, Cambridge, MA (URL: http://exac.broadinstitute.org), February 2017]; both parents and S1 were heterozygous, confirming the segregation of the mutation with congenital hypothyroidism in the family (Fig. 1). No additional mutations were detected in known causative CH genes.

### *In vitro* confirmation of the pathogenicity of the DUOX1 splice site mutation

**Figure 2.**
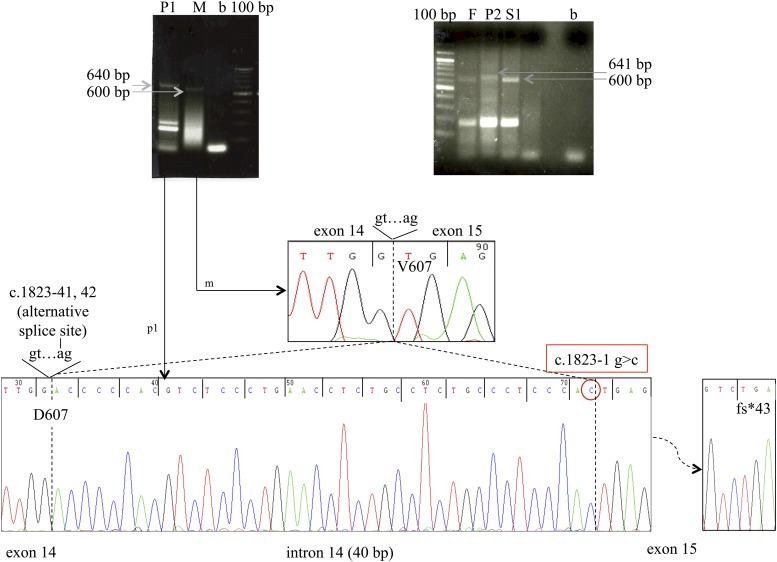
Complementary DNA amplification and sequencing from homozygous and heterozygous family members, demonstrating aberrant splicing of *DUOX1*. The electropherogram of the complementary DNAs and transcript sizes of the different *DUOX1* variants detected in the family members are shown. Exons are numbered with the first translated exon as exon 1. P1, P2: children with CH and homozygous DUOX1 c.1823-1G>C mutation. M, mother; F, father; S1, sister; all unaffected and heterozygous for the DUOX1 c.1823-1G>C mutation.

In the heterozygotes, a wild-type DUOX1 fragment of expected size (622 bp) was amplified from peripheral blood mononuclear cells. In contrast, in the homozygotes, a higher molecular weight band was detected and sequencing confirmed a 40-bp insertion in the 3′ portion of intron 14, indicating the activation of a cryptic acceptor site in intron 14 [r.(1823-40_1823-1ins; 1823-1G>C)] (Fig. 2). This alternative splicing generates a frameshift and a stop codon in exon 15 (p.Val607Aspfs*43) predicted to truncate DUOX1 within the transmembrane helices shortly after the peroxidase domain [Supplemental Fig. 1(B)]. In the heterozygotes, the high instability of this alternative splicing/nonsense transcript, derived from the mutant *DUOX1* allele, may have led to the preferential amplification of the correctly spliced wild-type transcript (Fig. 2).

### Further biochemical evaluation

Urine iodine measurements were not available at diagnosis, but subsequent spot measurements suggested mild (P2, 73.9 µg/L) to moderate (P1, 45.6 µg/L) iodine deficiency (RR 100 to 700 µg/L). Moderate iodine deficiency in association with double heterozygosity for *DUOX1* and *DUOX2* mutations (S1 and parents) did not result in hypothyroidism (urinary iodine: mother 39.2 µg/L; father 38.7 µg/L; S1 43.1 µg/L; RR 100 to 700 µg/L) (Fig. 1).

## Discussion

We report CH cases harboring a homozygous loss-of-function mutation in *DUOX1* (c.1823-1G>C), inherited digenically with a homozygous *DUOX2* nonsense mutation (c.1300 C>T, p. R434*) (3, 4). The tertiary structure of DUOX1 and -2 is summarized in Supplemental Fig. 1(B); aberrant splicing of DUOX1 (c.1823-1G>C) will generate a truncated protein (p.Val607Aspfs*43) lacking the C-terminal flavin adenine dinucleotide and NADPH binding domains and cytosolic Ca^2+^ binding sites (EF-hand motifs) [Supplemental Fig. 1(B)].

*In vitro* evaluation of a similarly truncated DUOX1 isoenzyme comprising amino acids 1 to 593 alone abolished H_2_O_2_-generating activity (5). Moreover, similar truncations in the highly homologous DUOX2 [p.Q686*, p.R701*, p.(G418fsX482);(IVS19-2A>C), p.S965fsX994] are associated with CH or severely impaired H_2_O_2_-generating activity *in vitro* (4, 6, 7). The c.1823-1G>C mutation would be predicted to generate a nonfunctional DUOX1 enzyme, and its digenic inheritance alongside the homozygous DUOX2 p.R434* will likely result in complete absence of functional DUOX isoenzyme in our patients.

It has been speculated that DUOX1 upregulation in the context of DUOX2 loss of function may at least partially compensate for defective H_2_O_2_ production. In support of this notion, the majority of reported biallelic *DUOX2* mutations, which are known to truncate the protein before the H_2_O_2_-generating domains, cause transient or mild permanent CH, despite presumably abrogating DUOX2 activity completely (8, 9) (Table 1). Direct comparison of biochemistry from reported cases with measurements made in our kindred is precluded by lack of T4 measurement in P2, and the fact that CH was diagnosed in P1 and her deceased sibling aged 6 months, rather than neonatally. However, in cases with biallelic truncating mutations, there is a broad spectrum of FT4 measurements at diagnosis, ranging from undetectable to 1.5 ng/dL, and partial iodide organification defects in all except one evaluated case. As well as mandating that other enzymes besides DUOX2 are capable of thyroidal H_2_O_2_ synthesis, this observation suggests heterogeneity in the efficiency of this compensatory process, likely due to either genetic or environmental modulators. Digenic, homozygous *DUOX2* and *DUOX1* mutations in our patients are associated with uncharacteristically severe CH; therefore, we speculate that inability of DUOX1 to compensate for defective H_2_O_2_ production may be contributing to disease severity. Unfortunately, the close chromosomal proximity of *DUOX1* and *DUOX2* mandates cosegregation of the two mutations, precluding evaluation of their individual contributions.

**Table 1.** **Table Summarizing Clinical Phenotype and Genotype Information for Published Cases Harboring Biallelic, Confirmed Truncating Mutations in DUOX2**

**Reference**	**Case**	**DUOX2 Mutation**	**bsTSH (mU/L)**	**vTSH (mU/L)**	**FT4 (ng/dL)**	**US**	**KClO_4_** **(%)**	**CH Duration**
Current cases	P1	p.[R434*];[R434*]	—	>100*^a^*	<0.09*^a^*	—	—	P
	P2	p.[R434*];[R434*]	—	>100*^b^*	—	G	—	P
Nicholas *et al.*, 2016 (10)	1	p.[ L1028Afs*3];[ L1028Afs*3]	—	55	—	N		—
Tan *et al.*, 2016 (11)	2	p.[K530*];[K530*]	14.76	86.06	0.76	G	—	T
	3	p.[K530*];[K530*]	111.45	>100	<0.4	N	—	T
	4	p.[K530*];[K530*]	20.25	>100	<0.4	G	—	T
	5	p.[K530*];[K530*]	122.66	23.9	0.92	N	—	MP
	6	p.[K530*];[Q202Rfs*93]	9.3	>100	<0.4	G	—	T
	7	c.647-656del10ins15/p.K530*	54.64	92.38	0.60	G	—	MP/T
	8	p.[K530*];[K530*]	14.05	9.58	0.92	G	—	T
	9	p.[K530*];[K1174Sfs*12]	11.88	>100	0.49	N	—	—
	10	p.[R701*];[K530*]	46.17	>100	0.43	G	—	MP/T
	11	p.[Q202Tfs*99];[K530*]	14.47	12.1	1.03	G	—	—
Fu *et al.*, 2016 (9)	12	p.[K530*];[K530*]	>8	>100	0.17	N	—	T
	13	p.[L1114Sfs*56];[K530*]*^c^*	>8	>100	0.32	N	—	T
Fu *et al.*, 2015 (12)	14	p.[L1114Sfs*56];[K530*]*^c^*	>8	>100	0.32	N	—	T
	15	p.[L1114Sfs*56;W301C];[K530*]	>8	>100	0.63	H	—	P
Muzza *et al.*, 2014 (2)	16	p.[Q202Tfs*99];[T522Pfs*64]	18	180	—	—	57	P
	17	p.[Q202Tfs*99];[T522Pfs*64]	21	130	—	—	66	P
Maruo *et al.*, 2008 (8)	18	p.[L479Sfs*2];[K628Rfs*10]	36.9	95.4	0.43	G	—	T
	19	p.[L479Sfs*2];[K628Rfs*10]	21.4	233	0.19	G	—	T
	20	p.[L479Sfs*2];[K628Rfs*10]	18.5	150	0.53	G	—	T
	21	p.[L479Sfs*2];[K628Rfs*10]	10	25.7	1.5	G	—	T
Varela *et al.*, 2006 (6)	22	p.[G418Efs*64];c.[2655-2A>C]	—	>100	<1*^d^*	G	60	P
	23	p.[G418Efs*64];c.[2655-2A>C]	—	>100	0.8*^d^*	G	68	P
Moreno *et al.*, 2002 (4)	24	p.[R434*];[R434*]	>50	1400	0.07	—	100	P

Abbreviations: bsTSH, blood spot screening TSH; G, goiter; H, hypoplastic; KClO_4_, perchlorate discharge; MP, mild permanent; N, normal; P, permanent; T, transient; US, ultrasound; vTSH, venous confirmatory TSH.

^a^ Biochemistry aged 6 months (P1).

^b^ Biochemistry aged 8 days (P2).

^c^ Compound heterozygosity assumed.

^d^ Total T4, μg/dL, normal range 5.98 to 13.9, measured aged 8 months (case 22) and 1 month (case 23). Normal ranges: FT4 ng/dL: Moreno *et al*., 0.9 to 2.3; Fu *et al*., 0.9 to 1.7; Maruo *et al*., 0.97 to 1.7; Tan *et al*., 0.9 to 2.28.

Urinary iodine was not measured contemporaneously with CH diagnosis in our kindred, and subsequent spot measurements did reveal mild–moderate iodine deficiency across the family, for which we cannot exclude a phenotypic contribution. Indeed, high dietary iodine intake in Japan is postulated to mitigate CH associated with DUOX2 mutations, accounting for the high frequency of transient CH in Japanese cases harboring biallelic mutations (Table 1). However, individuals in our kindred who were digenic for heterozygous *DUOX1* and *DUOX2* mutations remained euthyroid, despite moderate iodine deficiency, supporting a digenic, rather than environmental cause for the severe phenotype in the homozygous offspring.

It is noteworthy that the only other reported case harboring the homozygous *DUOX2* p.R434* mutation (also Turkish) is unique in manifesting both total iodide organification defect and uncharacteristically severe CH. Although the disease severity in both kindreds could reflect an intrinsic characteristic of the very proximal DUOX2 p.R434* mutation, this mutation is likely to be functionally identical to the Q202TfsX99, K530*, and T522PfsX64 truncating mutations, which will also truncate DUOX2 within the peroxidase-like domain before the first transmembrane region. Cases harboring such biallelic mutations have been associated with partial iodide organification defect and variable disease trajectory, including transient CH, suggesting that factors other than the p.R434* DUOX2 mutation itself are contributing to disease severity (6, 8, 9) (Table 1). Mutations in coding regions and intron–exon boundaries of *DUOX1* were excluded in the reported p.R434* mutation case, which argues against digenic inheritance of the same *DUOX1* mutation as a founder effect in the Turkish population. However, oligogenic variants in other known hitherto undiscovered CH-associated genes (including the noncoding regions of *DUOX1*) may be contributing to disease severity. Alternative phenotypic modulators could include polygenic factors specific to the Turkish ethnic background, or environmental iodine deficiency, because iodine status was not evaluated (4). No DUOX1-sequencing results are reported for other cases with permanent CH associated with biallelic truncating *DUOX2* mutations listed in Table 1, although variants in other CH-associated genes were occasionally sought (2, 6, 9, 10).

In the wider CH context, next-generation sequencing technologies are elucidating a role for oligogenicity in disease pathogenesis (10). We describe the first human cases with digenic *DUOX* mutations causing complete DUOX isoenzyme deficiency in the context of likely iodine deficiency. These individuals manifest severe CH, suggesting failure to compensate for defective thyroid H_2_O_2_ synthesis. Although limited subphenotype information prevents definitive ascertainment of the relative roles of the two mutations in the thyroid dysfunction, we hypothesize that inability of DUOX1 to compensate for DUOX2 deficiency contributes to disease severity in this kindred. Further studies are required to interrogate the role of upregulation of DUOX1 and alternative H_2_O_2_-producing enzymes in DUOX2-deficient cases and the contribution of variants in these genes to the phenotypic heterogeneity associated with *DUOX2* mutations.
