# Supplementary material for: Digenic DUOX1 and DUOX2 Mutations in Cases With Congenital Hypothyroidism
Source: J Clin Endocrinol Metab. 2017 Jun 16;102(9):3085–90. doi: 10.1210/jc.2017-00529 (PMC5587079; doi:10.1210/jc.2017-00529)
Supplement: Supplementary file 2 [file jc.2017-00529.sm1.docx]

**Supplementary Methods**

***DUOX1* sequencing**

The DNA template of the *DUOX1* gene was downloaded from the Ensembl database (ENSG00000137857) and the Sanger sequencing of the variant c.1823-1G>C was performed following PCR amplification of genomic DNA using specific primers:

*DUOX1* 15exF: ATTGCCAGGTAAGGAGCTGA

*DUOX1* 15exR: GGTGGAGGTGGGTAGCTGTA

Briefly, the PCR product was size-checked on 1% horizontal agarose gel and cleaned up using MicroCLEAN (Microzone, Haywards Heath, UK). The purified PCR product was sequenced in both forward and reverse directions using the ABI BigDye Terminator v3.1 Cycle Sequencing kit on an ABI Prism 3730 DNA Analyzer (Applied Biosystems, Warrington, UK). The sequence was downloaded using Chromas software and assessed manually for the presence of the alteration.

Variants are reported using nomenclature approved by Human Genome Variation Society (HGVS; [www.hgvs.org/mutnomen](http://www.hgvs.org/mutnomen) with nucleotide and exon numbering starting from the A (+1) of the translation initiation ATG codon of the *DUOX1* cDNA sequence RefSeq NM_ 175940.2).

**Whole exome sequencing**

Whole exome sequencing of P1 and P2 was undertaken using SureSelect Human All Exon 50Mb Kit (Agilent Technologies)and SOLiD™4 System (Applied Biosystems) as previously described (Sun 2012).

The mean coverage of the exons was 77 times. Sequence was aligned to the human reference genome build UCSC hg19 (Grch37) with the Burrows-Wheeler Aligner (Pitceathly 2009). To improve raw alignment BAMs for SNP calling, sequence was realigned around known (1000 Genomes pilot) indels and recalibrated base quality scores using GATK (DePristo 2011). SAMtools calmd was used to add Base alignment quality tags. BAMs for each sample were merged and duplicates marked using Picard. Variants (SNPs and indels) were called using SAMtools mpileup (0.1.17) (Li 2009) and GATK UnifedGenotyper (1.3.21) (McKenna 2010) and annotated with 1000 Genomes allele frequencies phase I integrated call set, dbSNP137 rsids, and earliest appearance in dbSNP. Functional annotation was added using Ensembl Variant Effect Predictor v2.8 against Ensembl 70. Shared homozygous variants with likely functional consequences with MAF <1% were then analyzed. Candidate genes were prioritised according to the following criteria:

• Genes expressed in thyroid tissue

• Genes with a recognised expressed sequence tag or mRNA

• Genes coding for proteins which have physiological roles in known-pathways which might be disrupted in CH

• Involvement of the gene/protein product in other known diseases

• Genes with available animal model systems

On the other hand, putative variants in candidate genes were evaluated by (i) segregation with disease status in family members, (ii) inclusion in reliable databases as ‘disease causing mutation’, (iii) evolutionary conservation, (iv) predicted effects on gene products, (v) scores with *in silico* prediction tools, and (vi) absence in >400 ethnically matched control chromosomes.

**In vitro studies of the *DUOX1* splice site mutation**

RNA was extracted from peripheral leukocytes of the Probands and unaffected relatives (PAXgene Blood RNA System, PreAnalytiX, Hombrechtikon, CH) and total RNA was reverse transcribed with Superscript reverse transcriptase II (Invitrogen Life Technologies Corp., Carlsbad, CA) using a random hexamer mixture as primers. Each cDNA was amplified by polymerase chain reaction (PCR) using the following exonic primers spanning exons 14-18: DUOX1 14exF: ACCATCGGGACCCTCTGT and DUOX1 18exR: CCTGCTCAGCTCACAGGT. PCR products were subjected to 2% agarose gel electrophoresis, excised and purified using QIAquick Gel Extraction Kit (Qiagen Sciences, Germantown, MD) and directly sequenced.

**Supplemental References**

Sun Y, Bak B, Schoenmakers N, van Trotsenburg AS, Oostdijk W, Voshol P, Cambridge E, White JK, le Tissier P, Gharavy SN, Martinez-Barbera JP, Stokvis-Brantsma WH, Vulsma T, Kempers MJ, Persani L, Campi I, Bonomi M, Beck-Peccoz P, Zhu H, Davis TM, Hokken-Koelega AC, Del Blanco DG, Rangasami JJ, Ruivenkamp CA, Laros JF, Kriek M, Kant SG, Bosch CA, Biermasz NR, Appelman-Dijkstra NM, Corssmit EP, Hovens GC, Pereira AM, den Dunnen JT, Wade MG, Breuning MH, Hennekam RC, Chatterjee K, Dattani MT, Wit JM, Bernard DJ. [Loss-of-function mutations in IGSF1 cause an X-linked syndrome of central hypothyroidism and testicular enlargement.](http://www.ncbi.nlm.nih.gov/pubmed/23143598) *Nat Genet*. 2012; **44**:1375-81.

Pitceathly RDS, Rahman S, Wedatilake Y, Polke JM, Cirak S, Foley AR, Sailer A, Li H, and Durbin R.. Fast and accurate short read alignment with Burrows- Wheeler transform. *Bioinformatics* 2009; 25:1754–60

DePristo MA, Banks E, Poplin R, Garimella KV, Maguire JR, Hartl C, Philippakis AA, del Angel G, Rivas MA, Hanna M, et al. A framework for variation discovery and genotyping using next-generation DNA sequencing data. *Nat Genet*. 2011; 43:491–8.

Li H, Handsaker B, Wysoker A, Fennell T, Ruan J, Homer N, Marth G, Abecasis G, and Durbin R; 1000 Genome Project Data Processing Subgroup. The Sequence Alignment/Map format and SAMtools. *Bioinformatics* 2009; 25:2078–9.

McKenna A, Hanna M, Banks E, Sivachenko A, Cibulskis K, Kernytsky A, Garimella K, Altshuler D, Gabriel S, Daly M, and DePristo MA. The Genome Analysis Toolkit: a MapReduce framework for analyzing next generation DNA sequencing data. *Genome Res*. *2010*; 20:1297–303.
